# Supplementary figures and images for: Magnetic resonance angiography in diagnostic long-term follow-up of primary patency of the MOTIV drug-eluting bioresorbable vascular scaffold in the region below the knee: 5 years of experience
Source: PLoS One. 2025 Jan 24;20(1):e0313696. doi: 10.1371/journal.pone.0313696 (PMC11761161; doi:10.1371/journal.pone.0313696)

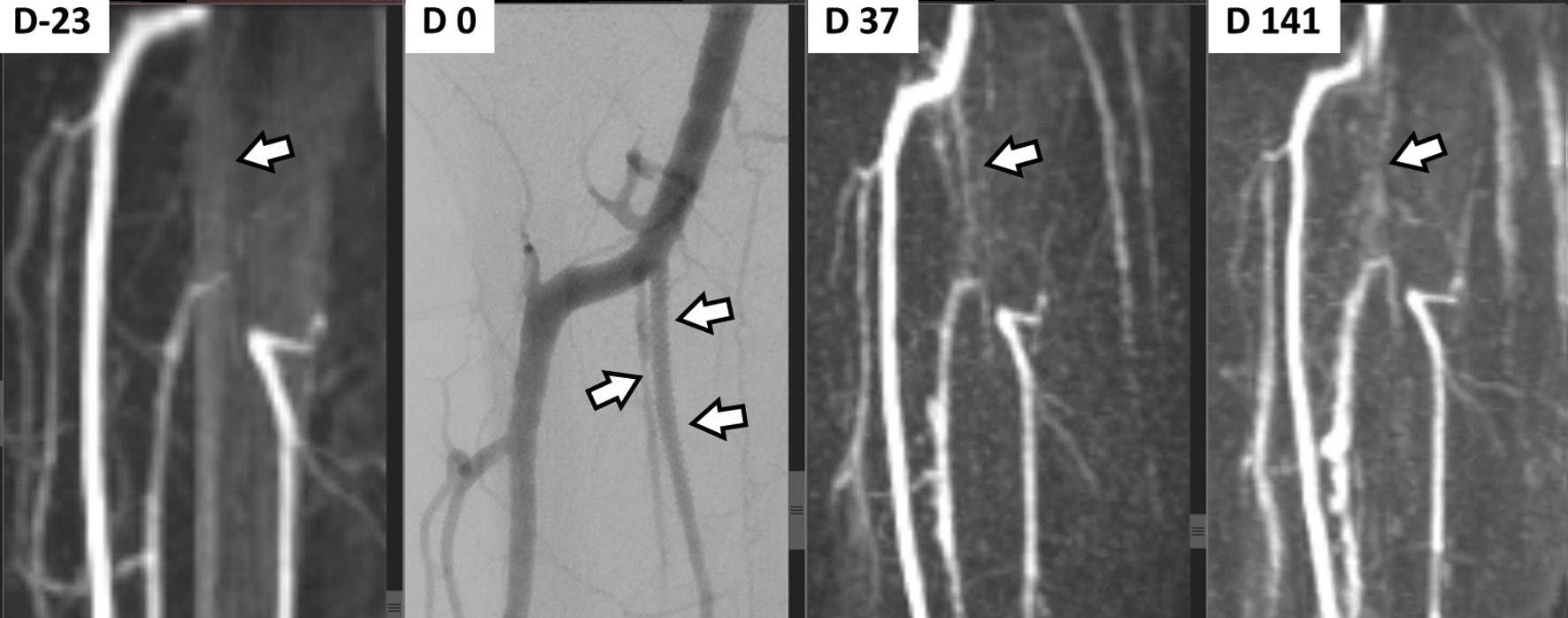

Supplement: S1 Fig — The occlusion was confirmed also 141 days later. (white arrows mark the position of the deBVS). (TIF) [file pone.0313696.s002.tif]
