# Supplementary material for: Magnetic resonance angiography in diagnostic long-term follow-up of primary patency of the MOTIV drug-eluting bioresorbable vascular scaffold in the region below the knee: 5 years of experience
Source: PLoS One. 2025 Jan 24;20(1):e0313696. doi: 10.1371/journal.pone.0313696 (PMC11761161; doi:10.1371/journal.pone.0313696)
Supplement: Attachment — Submitted filename: Response to Reviewers.doc [file pone.0313696.s003.doc]

# Dear Editor!

Thank You for the opportunity to share our results with the scientific community of the Journal PLoS. Please find enclosed the point-by-point statements of the requested issues.

# Journal Requirements:

1^st^ point) The link proposed in Your letter is not valid (URL: https://journals.plos.org/plosone/s/file?id=wjVg/ PLOSOne_formatting_sample_main_body.pdf; accessed last: 2024.09.18). Instead we re-checked the submission guide lines (URL: <https://journals.plos.org/plosone/s/submission-guidelines>; accessed last: 2024.09.18). For references, as we use EndNote, the most recent style was used. We found some deviations, which were corrected. We hope that this fixes the problems during the review process.

2^nd^ point) According to the standards published by PLoS (URL: <https://journals.plos.org/plosone/s/materials-and-software-sharing>; accessed last: 2024.09.18) software development was no central part of the manuscript. We employed cran R to evaluate our measurements using only standard functions approved by the publisher. All statistical functions used are publicly available with the cran R kernel or the packages already cited in the original paper.

3^rd^ point) Sorry, we though that this was a direct question. Yes, we would like to state in the new submission: 'The authors have declared that no competing interests exist.'

4^th^ point) Actually, all relevant data are already within the manuscript. However, a Supporting Information-file meeting the “minimal data set”-policy of PLoS was added, not least in any case this was necessary to clearly present all measurements.

5^th^ point) The requested captions were added at the end of the manuscript.

# Comments to Reviewers

## Reviewer 1

1^st^ point) We thank reviewer 1 for this comment. These abbreviations have the same meaning. During replacement of older versions, we unintentionally skipped table 2 and figure 4, when we tried to find a most convenient abbreviation for this measure. Now the abbreviation: "NIL-S_deBVS_" is used throughout the paper. Figure 4 was also corrected.

2^nd^ point) We provided a supporting information csv-sheet that comprehensively shows all data. Table 2 was rigorously reworked (see also comments to reviewer 2) and should be easier to read now. Our intention was to present the full set without any restrictions, which was obviously too confusing. However, all values of the original submission and the revision can be calculated from the supporting information csv-sheet.

3^rd^ point) We inserted a supporting information figure demonstrating an early occlusion of the deBVS. The corresponding measurements (p19) can be derived from the supporting information csv-sheet. We hope this meets the request.

## Reviewer 2

1^st^ point) We agree that the sample size is small. Therefore, this was pointed out in the limitations. However, we disagree with reviewer 2 that the conclusions lack reliability, since the compatibility of the Tyrocore®-stent with MRI was also proven in-vitro and could be reliably confirmed with MR-A in-vivo. If this deBVS significantly annihilated the regional MR-signal, we would not have been able to judge a single patient correctly. On the contrary, we did not encounter a diagnostic restriction in any of our cases. Thus, from a statistical point of view we deal with an extremely strong positive statistical effect in favour of unrestricted MRI after implantation of the investigated deBVS. This can be tested already in a small number of cases. Though for statistical purposes a bigger number of cases would have been desirable, one cannot conclude that the direct depiction of patency of vessel segments treated with the deBVS would be not feasible. As diagnostic MRI after placement of this deBVS seems preserved, of course, we reported the number of stents censored and occluded. We do not claim that our retrospectively estimated probability of primary patency is precise enough to hold against future prospective data, but our findings provide a first and reasonable impression that this MR-A assessable deBVS may perform not worse compared to published data of metallic drug eluting stents in the BTK-region. Therefore, the conclusion that the use of a deBVS comparable to the tested one together with MR-A seems recommendable, because diagnostic and treatment options appear to be preserved, may indeed be derived even from our small sample.

2^nd^ point) We agree that presenting the complete data in a clear and well-arranged fashion in the manuscript is difficult. A full step-by-step explanation of the collection and inclusion process of all data is now inserted at the end of the 'Patients'-subsection in the 'Methods'-chapter (revision: lines 96-109). Additionally, the flow chart demonstrating the patient collection (figure 1), was also revised and adapted to the inserted explanation. We hope that this renders the data collection easier to read.

3^rd^ point) There was not at all a comparison between CT-A and MR-A reported in our paper. Just to get a better estimate of the PP-probability of the investigated deBVS, due to the small number of cases, we censored one patient receiving CT-A only for the Kaplan-Meyer estimates. This was clearly stated in the original submission. Nevertheless, this is now explained in more detail in the revision (revision: lines 109-113).
On the other hand, according to the strict open data policy of PLoS, we could not simply conceal the patency data of this patient, because we very well knew the state of the deBVS. This is quite different to patients lost to follow-up, where the patency state is not known at all and who, therefore, can be simply excluded. The estimated PP-probability was only used to underline the recommendation that it is worth to consider MR-A instead of diagnostic modalities exposing the patients to radiation, because these scaffolds seem to perform comparable to metallic stents but do not restrict in MR-A like the metallic ones. We do not believe that this skews the data or any of our conclusions, moreover, as this was always clearly communicated to the reader. We hope that this is also acceptable.

4^th^ point) There is absolutely no rationale to sacrifice the life of even a single animal to convince anybody that MR-A can be used with the investigated deBVS, since, as shown in our paper, we have enough evidence in humans already and experimental data from a specimen. We are bewildered that PLoS, given the data of our paper, can agree with this suggestion, which clearly contradicts the 3-R-principle of animal experiments. Of course, taking this into account, an ethics committee in Europe would not accept such experiments and, thus, no animal experiments can or will be conducted.

5^th^ point) We deleted this and a few other abbreviations, which did not conform to PLoS-format standards.

6^th^ point) We recognise that table 2 may be confusing and, therefore, it was rigorously revised. We hope the actual one is easier to read now.

Yours respectfully,

Christian Nasel
